# 

**Published:** 1832-11

**Authors:** 


### MONTHLY LIST OF MEDICAL BOOKS.

[*Medical Works cannot be entered on this List unless a copy be sent for the purpose, the titles of Books having frequently been sent to us as published which have not appeared for weeks, or even months, after.*]

The Cyclopædia of Practical Medicine. Edited by JOHN FORBES, M.D., A. TWEEDIE, M.D., and JOHN CONOLLY, M.D. Part X. (published in Monthly Parts,) October 1832,) Containing *Headach* (continued), by Dr. BURDER; *Heart, Diseases of the,* Dr. HOPE; *Heart, Displacement of the,* Dr. TOWNSEND; *Heart, Fatty and Greasy Degenerescence of the,* Dr. HOPE; *Hematemesis,* Dr. GOLDIE; *Hæmoptysis,* Dr. LAW; *Hemorrhage,* Dr. WATSON; *Hereditary Transmission of Disease,* Dr. BROWN; *Herpes,* Dr. A. T. THOMSON; *Hiccup,* Dr. ASH; *Hooping Cough,* Dr. JOHNSON; *Hydatids,* Dr. KERR; *Hydrocephalus,* Dr. JOY; *Hydropericardium,* Dr. DARWELL; *Hydrophobia,* Dr BARDSLEY.—Royal 8vo. Sherwood, Gilbert, and Piper; and Baldwin and Cradock, London.

The Sources of Health and Disease in Communities; or, Elementary Views of 'Hygiêne,' illustrating its Importance to Legislators, Heads of Families, &c. By HENRY BELINAYE, Esq., Surgeon Extraordinary to her Royal Highness the Duchess of Kent, &c.—8vo. pp. 261. Treuttell and Würtz, and Riehter, London.

A Practical Treatise on Cholera, as it has appeared in various parts of the Metropolis. By ALEXANDER TWEEDIE, M.R.C.S., Resident Medical Officer to the City of London Cholera Hospital in Abchurch lane; and CHARLES GAZELEE, M.R.C.S, Surgeon to the Marshalsea Prison.—8vo. pp. 79. Elder and Co., London.

The Anatomy of the Horse, embracing the Structure of the Foot. By WM. PERCIVALL, M.R.C.S., Veterinary Surgeon in the First Life Guards; author of "Lectures on the Veterinary Art," and co-editor of "The Veterinarian."—8vo. pp. 454. Longman, London.

On the Influence of Physical Agents on Life. By W. F. EDWARDS, M.D., F.R.S.. Member of the Royal Academy of Sciences and Royal Academy of Medicine of Paris, of the Philomathic Society of the same city, and of the Medical Society of Dublin, &c. Translated from the French, by Dr. HODGKIN and Dr. FISHER. To which are added, in the Appendix, some Observations on Electricity, by Dr. EDWARDS, M. POUILLET, and LUKE HOWARD, F.R.S.; on Absorption, and the Use of the Spleen, by Dr. HODGKIN; on the Microscopic Characters of the Animal Tissues and Fluids, by J. J. LISTER, F.R.S., and Dr. HODGKIN; and some Notes to the Work of Dr. EDWARDS.—8vo. pp. 488. Highley, London.

Elements of Materia Medica and Therapeutics; including the recent Discoveries and Analyses of Medicines. By ANTHONY TODD THOMSON, M.D., F.L.S. and G.S., Professor of Materia Medica and Therapeutics, and of Medical Jurisprudence, in the University of London; Member of the Royal College of Physicians, &c. Vol. I.—8vo. pp. 747. Longman, London.

Nos. I. and II. of the Flowering Plants of Oxfordshire. Edited by WM. BAXTER, Curator of the Botanic Gardens in the University of Oxford.

No. I. of Medical Botany: or, Illustrations and Descriptions of the Medicinal Plants of the London, Edinburgh, and Dublin Pharmacopœias, including a popular and scientific Account of Poisonous Vegetables indigenous to Great Britain, with Figures drawn and coloured from Nature. By JOHN STEPHENSON, M.D. F.L.S., and J. M. CHURCHILL, F.L.S. New Edition, edited by GILBERT T. BURNETT, F.L.S., Professor of Botany in King's College, London, &c. —John Churchill, London.

The Nature and Treatment of the Epidemic Cholera. By Bobert Venables, A.M.: with an Appendix.—Peter Reid, Wick, North Britain.

An Introduction to Botany. By John Lindley, F.R.S. L.S. G.S. &c., and Professor of Botany in the University of London. With six Copper-plates and numerous Wood-Engravings.—Longman and Co., London.

---

### APOTHECARIES' HALL.

Names of Gentlemen to whom the Court of Examiners have granted Certificates:

OCT. 4.

Henry Crooks, of London
Robert Eminson, Scotter, Lincolnshire
Edward Murray, London
John Martin, Merthyr Tidvil
John Nicholls, Frome

OCT. 11.

John Spark

OCT. 18.

Lees Broadbent, of Ashton under Lyne
Samson Carey
Thomas James Cottle
John Hilliard

OCT. 25.

Philip Henry Dicker, of Lewes
Stephens Jennings Goodfellow, Falmouth
Arthur Martin
Peter Marshall, London
Thomas Peregrine, London
Francis Taylor, Hull.

---

